# Primary cells derived from Tuberous Sclerosis Complex patients show autophagy alteration in the haploinsufficiency state

**DOI:** 10.1590/1678-4685-GMB-2020-0475

**Published:** 2021-10-01

**Authors:** Clévia Rosset, Mariane da Cunha Jaeger, Eduardo Filippi-Chiela, Larissa Brussa Reis, Ivaine Taís Sauthier Sartor, Cristina Brinckmann Oliveira, Caroline Brunetto de Farias, Rafael Roesler, Patricia Ashton-Prolla

**Affiliations:** 1 Hospital de Clínicas de Porto Alegre (HCPA), Centro de Pesquisa Experimental, Laboratório de Medicina Genômica, Porto Alegre, RS, Brazil.; 2 Universidade Federal do Rio Grande do Sul (UFRGS), Programa de pós-graduação em genética e biologia molecular, Porto Alegre, RS, Brazil.; 3 Hospital de Clínicas de Porto Alegre (HCPA), Centro de Pesquisa Experimental, Laboratório de Câncer e Neurobiologia, Porto Alegre, RS, Brazil.; 4 Universidade Federal do Rio Grande do Sul (UFRGS), Faculdade de Medicina (Famed), Programa de pós-graduação em gastroenterologia e hepatologia, Porto Alegre, RS, Brazil.; 5 Hospital de Clínicas de Porto Alegre (HCPA), Serviço de Genética Médica, Porto Alegre, RS, Brazil.; 6 Instituto do Câncer Infantil (ICI), Porto Alegre, RS, Brazil.; 7 Universidade Federal do Rio Grande do Sul (UFRGS), Instituto de Ciências Básicas da Saúde (ICBS), Departamento de Farmacologia, Porto Alegre, RS, Brazil.; 8 Universidade Federal do Rio Grande do Sul (UFRGS), Departamento de Genética, Porto Alegre, RS, Brazil.

**Keywords:** Autophagy, mTOR inhibitors, neurocutaneous disorder, Rapamycin, Tuberous Sclerosis Complex

## Abstract

Tuberous sclerosis complex (TSC) is an autosomal dominant cancer predisposition disorder caused by heterozygous mutations in *TSC1* or *TSC2* genes and characterized by mTORC1 hyperactivation. TSC-associated tumors develop after loss of heterozygosity mutations and their treatment involves the use of mTORC1 inhibitors. We aimed to evaluate cellular processes regulated by mTORC1 in TSC cells with different mutations before tumor development. Flow cytometry analyses were performed to evaluate cell viability, cell cycle and autophagy in non-tumor primary TSC cells with different heterozygous mutations and in control cells without *TSC* mutations, before and after treatment with rapamycin (mTORC1 inhibitor). We did not observe differences in cell viability and cell cycle between the cell groups. However, autophagy was reduced in mutated cells. After rapamycin treatment, mutated cells showed a significant increase in the autophagy process (p=0.039). We did not observe differences between cells with distinct *TSC* mutations. Our main finding is the alteration of autophagy in non-tumor TSC cells. Previous studies in literature found autophagy alterations in tumor TSC cells or knock-out animal models. We showed that autophagy could be an important mechanism that leads to TSC tumor formation in the haploinsufficiency state. This result could guide future studies in this field.

## Introduction

Tuberous sclerosis complex (TSC) is an autosomal dominant cancer predisposition disorder with a birth incidence estimated to be 1 in 6000, with an equal male/female distribution (Osborne et al., 1991). It is caused by mutations in either of two genes, *TSC1* or *TSC2*, which code for hamartin and tuberin, respectively (Povey et al., 1994). TSC is a complex disorder with many features, ranging from mild to severe symptoms. It is characterized by neuropsychiatric disorders and multiple hamartomas, mainly in the brain, kidneys, heart, and lungs (Crino et al., 2006)_._ Hamartomas develop according to the Knudson two hit hypothesis, which means that the loss of heterozygosity (LOH) is necessary to their occurrence (Knudson, 1971).

Hamartin and tuberin form a heterodimeric complex that acts in the suppression of mammalian target of rapamycin (mTOR) in mTORC1 complex (Soucek et al., 1997). mTORC1 is a highly conserved serine/threonine kinase that is a master regulator of cell growth, proliferation, survival, angiogenesis, autophagy, cellular senescence and immune reactions through its effector proteins, 4E-BP1, p70, S6K1 and eIF4E (Napolioni and Curatolo, 2008). In humans, mTORC1 is constitutively activated in the presence of growth factor and nutrients and acts as a master switch of cellular catabolism and anabolism (Dennis et al., 2001; Shaw and Cantley, 2006). When growth factors or nutrients are lacking, catabolic processes such as fatty acid oxidation or autophagy are induced to provide a constant supply of nutrients and maintain ATP production (Deberardinis et al., 2008). Thus, mTORC1 is a major negative regulator of autophagy by regulating the Atg1/ULK1 complex. In parallel to autophagy induction, the inactivation of mTORC1 inhibits cell growth (Balgi et al., 2009; Hung et al., 2012).

Mutations in either *TSC1* or *TSC2* can lead to chronic activation of mTORC1 and dysregulation of the aforementioned biological processes. TSC-associated tumors treatment with mTORC1 inhibitors has been proposed with variable responses (Franz, 2011, 2013; Franz *et al.*, 2013; Krueger et al., 2013; Dill et al., 2014; Muzic et al., 2014). mTORC1 inhibitors could restore the biological processes that are altered due to mTOR hyperactivation. Many studies that evaluate cell growth, proliferation, survival, and autophagy in TSC use knock-out and/or knock-in cell lineages and animal models, or primary cells from TSC tumors (Alayev et al., 2014; Valianou et al., 2015; Campos et al., 2016; Taneike et al., 2016; Carroll et al., 2017; Yang et al., 2018). However, the study of heterozygous non-tumor TSC cells without LOH is necessary to observe which biological processes are altered before tumor formation and are more important in TSC tumorigenesis. The knowledge of these processes could guide the development of novel targeted therapies and possibly prevent tumor formation. In this sense, this study seeks to evaluate which processes regulated by mTORC1 could be altered in TSC cells before tumor occurrence in patients with different *TSC* mutations.

## Material and Methods

### Samples

Biopsies (6 mm) of normal-appearing skin were obtained from five patients diagnosed with TSC according to clinical criteria (Roach et al., 1998) and with a mutation in *TSC1* or *TSC2* genes, previously identified by a customized Next Generation Sequencing panel and Multiplex Ligation Probe-dependent Amplification (Rosset et al., 2017). Table 1 shows the mutation profile of the five TSC patients. In addition, biopsies from two individuals without *TSC* mutations were obtained. The study was approved by the institutional review board (Comitê de Ética em Pesquisa do Hospital de Clínicas de Porto Alegre; GPPG 2015-0049) and experiments were carried out in accordance with the relevant guidelines and regulations. All individuals signed a specific written informed consent for this study.

**Table 1 -:** Mutation analysis of TSC patients included in this study.

Sample	Inheritance	Gender/Age at biopsy	Coding change	Amino acid change	Gene/Position	Mutation type	Classification^*^
Patient 1	Familial	F/48y	c.338T>A	p.Leu113Ter	*TSC1/* Exon 5	Nonsense	Pathogenic
Patient 2	Familial	F/53y	c.2074_2075insCTCC	p.Arg692fs^*^15	*TSC1/* Exon 17	Frameshift insertion	Pathogenic
Patient 3	Sporadic	F/18y	c.1008T>G	p.Tyr336Ter	*TSC2/* Exon 11	Nonsense	Pathogenic
Patient 4	Familial	F/18y	c.724delinsTCCT	p.Thr242Ser_Ser243del	*TSC2/* Exon 8	In frame delins	VUS
Patient 5	Familial	F/40y	c.4375C>T	p.Arg1459Ter	*TSC2/* Exon 34	Nonsense	Pathogenic

VUS = Variant of Uncertain Significance. ^*^According to the American College of Medical Genetics and Genomics (ACMG) Guidelines.

### Cell culture

Skin biopsies were used to establish primary fibroblast cultures. Briefly, skin samples were transferred into a 150-mm sterile dish containing HAM-F10 medium. The dermis was dissected from the rest of the skin (epidermis, subcutaneous tissue, vascular structures) using a scalpel. The dermis was cut into small pieces and about ten fragments were placed on the bottom of a 25 cm^2^ culture bottle separated from one another and dried for 15 minutes. HAM-F10 culture medium (3 mL) was added to cover the tissue pieces and cultures were placed into a humidified incubator at 5% CO_2_ and 37 °C and maintained in HAM-F10 medium with 1% penicillin-streptomycin and 15% fetal bovine serum (Gibco Laboratories, USA). Fibroblast migration out of tissue fragments were regularly monitored using an inverted microscope. This outgrowth method relies on the capacity of fibroblasts to migrate out of the skin and adhere to the surface of the culture vessel. This method has the advantage that the migrating cells are highly enriched in fibroblasts. When 80% confluence was obtained (passages 3-7), fibroblasts were trypsinized and expanded until five flasks. Subsequently, fibroblasts from each individual were transferred to a 12-well culture plate in triplicates, resulting in 3 plates and 36 wells per individual. Twenty-four hours after seeding, 12 of the 36 wells were treated with 10nM of the mTOR inhibitor rapamycin (R0395, Sigma Aldrich, Rehovot, Israel), the dose that corresponds to target therapeutic level (10 ng/mL) (Weidman et al., 2015), 12 were supplemented with the rapamycin vehicle dimethyl sulfoxide (DMSO), and 12 were supplemented with HAM-F10 medium (no treatment and no vehicle). Fibroblasts from each of the 36 wells were collected after 48 h (rapamycin half-life in plasma) and directly counted using trypan blue in Neubauer chamber.

### Cell viability evaluation

Three treated, three DMSO and three no treatment and no vehicle wells (one well from each of the 12-well plates) from each individual were centrifuged (7 min, 2100 rpm) and washed with phosphate buffered saline (PBS) 1x three times. Subsequently, cells were pooled into one tube and resuspended in PBS and propidium iodide (1 μg/μL; Sigma, Rehovot, Israel), and then analyzed in Attune Flow Cytometer (ThermoFisher Scientific, USA). Student-t test was performed for statistical analysis (considering p<0.05).

### Cell cycle evaluation

Six treated, six DMSO and six no treatment and no vehicle wells (two wells from each of the 12-well plates) from each individual were centrifuged (7 min, 2100 rpm) and washed with PBS 1x three times. Subsequently, cells were pooled into one tube, lysated and stained in a hypotonic stain buffer (sodium citrate 3.6 nM, propidium iodide 50 μg/mL, Triton X-100 0.1% and water to a final volume of 500 μLl). Cell cycle distribution was analyzed in Attune Flow Cytometer (ThermoFisher Scientific, USA) and Student-t test was performed for statistical analysis (considering p<0.05).

### Autophagy evaluation

Three treated, three DMSO and no treatment and no vehicle wells (one well from each of the 12-well plates) from each individual were centrifuged (7 min, 2100 rpm) and washed with PBS 1x three times. Subsequently, cells were resuspended in 500 μL HAM-F10 medium and incubated for 15 minutes with acridine orange solution (1 μg/mL). The percentage of autophagic cells was measured in Attune Flow Cytometer (ThermoFisher Scientific, USA). Student-t test was performed for statistical analysis (considering p<0.05).

## Results

**Figure 1 -:**
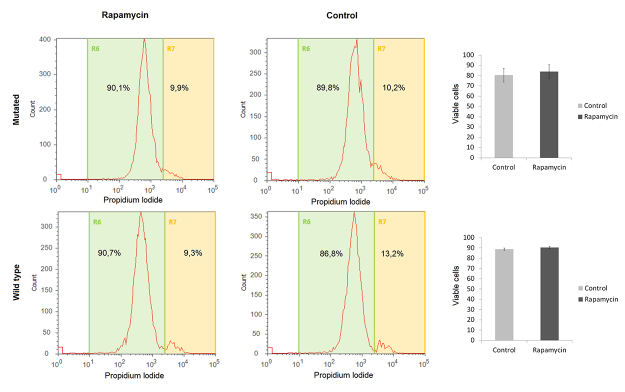
Cell viability analyses. Cells were stained with propidium iodide and submitted to Attune Cytometer analysis. Viable cells (%) of a representative mutated and WT samples are shown in the left panel. The average percentage of viable cells in mutated and wild-type groups is shown in the right panel.

**Figure 2 -:**
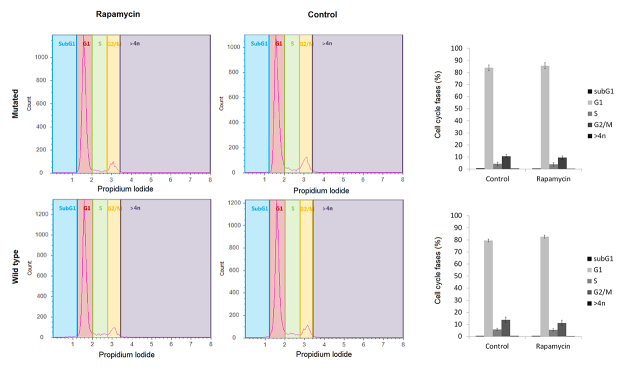
Cell cycle analyses. Cells were lysed, stained in a hypotonic stain buffer containing propidium iodide and submitted to Attune Cytometer cell cycle analysis. The quantification of the different cell cycle phases in a representative mutated and WT samples is shown in the left panel. The average percentage of cells in each cell cycle phase in mutated and WT cells is shown on the right.

**Figure 3 -:**
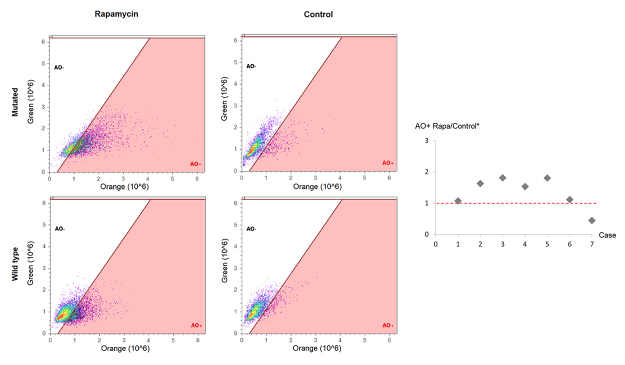
Autophagy analyses. Cells were incubated with acridine orange solution and submitted to Attune Cytometer analysis. Percentage of acridine orange positive (AO+) and acridine orange negative (AO-) cells of representative samples is shown on the left. The ratio of autophagic cells in wells treated and untreated with rapamycin for each case is shown in the right panel (cases 1-5 are mutated and cases 6-7 are wild type). There was an increase in the number of autophagic cells after rapamycin treatment in mutated cells (p=0.039)^*^.

We evaluated cell viability, cell cycle distribution and autophagy in heterozygous *TSC1* or *TSC2* mutated and wild-type fibroblasts, before and after treatment with rapamycin. We did not find differences in fibroblast growth rate and morphology between patients with and without TSC mutations, or in treated and non-treated cells in culture plates. Also, we did not observe differences in cell viability between these groups by flow cytometry. Figure 1 shows the percentage of viable cells in mutated and wild-type cells, treated with vehicle or rapamycin. This was strengthened by a cell counting experiment with Trypan blue. Cell cycle distribution of different groups is shown in Figure 2. There was no significant difference in the percentage of subG1 population in both mutated and wild-type groups, before and after treatment with rapamycin. Also, no statistical differences were observed in the average percentage of other cell cycle populations between groups. Finally, the percentage of positive and negative cells for acridine orange from mutated and wild-type wells treated with vehicle or rapamycin are shown in a representative plot in Figure 3 (left). The ratio of autophagic cells in wells treated and untreated with rapamycin for each case is shown in Figure 3 (right). The cells with *TSC* gene mutations showed a reduced number of acridine orange positive cells, an initial autophagy marker, compared to wild-type cells. After treatment with rapamycin, there was a statistically significant increase in the number of acridine orange positive cells in the mutated group compared to the wild-type group (p = 0,039). The number of acridine orange positive cells for each condition is shown in Table S1. We could not observe a large effect of rapamycin treatment. However, we emphasize that these results are important to stimulate future studies using heterozygous TSC cells to evaluate the autophagy pathway. We found for the first time that autophagy is already altered in heterozygous mutant TSC non-tumor cells and this alteration could be an initial mechanism to lead TSC tumor formation. Cell viability, cycle distribution, and autophagy were not different in cells with and without vehicle (DMSO). Also, we did not observe differences between cells with different *TSC* mutations.

## Discussion

In the recent years, efforts have been made to elucidate TSC phenotypic variability, to comprehend the large spectrum of *TSC1/2* mutations, and to clarify the mechanisms of tumor formation by focusing on mTORC1 and its related pathways. A significant portion of TSC patients using mTOR inhibitors do not achieve clinical remission and severe adverse reactions may also occur (Bissler et al., 2016). However, mTOR inhibitors are currently the unique treatment option and they are useful in experimental models to evaluate the restoration of the biological processes that are altered due to mTOR hyperactivation. Considering the number of different cellular processes regulated by mTORC1, tumor formation in TSC may involve multiple mechanisms. To date, studies that evaluated cell growth, proliferation, survival, and autophagy in TSC used knock-out and/or knock-in cell lineages and animal models, or primary cells from TSC lesions/tumors (Alayev et al., 2014; Valianou et al., 2015; Campos et al., 2016; Taneike et al., 2016; Carroll et al., 2017; Yang et al., 2018). TSC animal models have contributed to discoveries regarding disease consequences, including in brain development and function. However, there are important differences between TSC phenotype of human heterozygous TSC patients and heterozygous animal models, which have more subtle phenotypes (Afshar Saber and Sahin, 2020). Cell lineages and primary cells from lesions and tumors may not be the best model to evaluate which cellular processes are altered before tumor formation because in general these cells suffer LOH and are not heterozygous. Therefore, in order to evaluate which processes regulated by mTORC1 could be altered in TSC cells before tumor development, we evaluated heterozygous non-tumoral TSC cells without LOH to observe which biological processes are more important in initiation of TSC tumorigenesis.

Cell cycle progression and cell viability were not altered before tumor formation in our analyses. Indeed, this could be expected since loss of one *TSC1* or *TSC2* allele does not seem to severely affect embryonic or post-natal development in TSC patients. However, our goal was to evaluate if even small differences between patients and controls were present in cell cycle and survival and if these could have a role in tumor formation. Moreover, we analyzed cell death after 48 hours of treatment. This period of time may not be enough to induce cell death. However, to prevent variability, we evaluated the three mechanisms at the same time (rapamycin half-life in plasma), which could be enough to activate cell death in cells. In previous studies from medical literature, mTOR inhibition resulted in an increase in the turnover of cyclin D1 and a decrease in the elimination of the cyclin dependant kinase inhibitor P27 in fibroblast lineages (Takuwa et al., 1999). In another study, silencing *TSC2* in Rat1 fibroblasts shortened the G1 phase of the cell cycle, favouring cell cycle entry (Soucek et al., 1997). In a study with *TSC2* expressing and *TSC2* null cell lines, no differences in percentage of cells in cell cycle subsets were found after treatment with rapamycin (Makovski et al., 2014). None of the previous studies found in literature were conducted in primary human non-tumoral TSC cells.

We observed a reduction in the number of acridine orange positive cells in flow cytometry analyses of mutated TSC cells in comparison with wild-type cells. After treatment with rapamycin, the increase in the number of acridine orange positive cells is more significant in mutated cells than in the wild-type cells (p=0.039), indicating that autophagy is re-established by mTORC1 inhibition. Our methodology using acridine orange and flow cytometry analysis evaluates the final step of autophagy, which means that the process is actually occurring in the cell. As we are interested in the final stage of autophagy (autolysosomes), we used acridine orange to evaluate this mechanism. Acridine orange is a hydrophobic molecule that emits green fluorescence in neutral pH environments. Within acidic vesicles (autolysosomes), it becomes protonated and trapped within the organelle, emitting bright red fluorescence and thus showing the number of autolysosomes (the final stage of autophagy which we are interested in). This allows an objective quantification of the late step of autophagy in single cells, in contrast to quite all other autophagy markers which are semi-quantitative (e.g. western blot or immunohistochemistry) or subjective. Properties and specificities of acridine orange labeling were previously shown as a marker of the late stage of autophagy (Thomé et al., 2016).

Autophagy is increasingly recognized to play a critical role in tumor development and cancer therapy (Mizushima et al., 2008). mTORC1 is an important negative regulator of macroautophagy. It induces autophagy in response to reduced growth factor signalling, starvation, and other metabolic and genotoxic stresses which leads to the formation of phagophores, inside which the lysosomal hydrolases degrade organelles and intracellular proteins (Tooze and Schiavo, 2008; Jung et al., 2010). During physiological conditions, the phagophore formation is inhibited by mTORC1, since it directly interacts with and phosphorylates the Ulk1 kinase complex (Ulk1-Atg13-FIP200-Atg101) which is required for the initiation of autophagy (Hosokawa et al., 2009). In situations of bioenergetics stress, the Ulk1 complex is released from mTOR, thereby allowing it to associate with the membranes from which phagophores are formed (Mizushima and Levine, 2010). In this way, autophagy promotes the survival of established tumors by supplying metabolic precursors during nutrient deprivation; however, excessive autophagy has been associated with cell death (Rabinowitz and White, 2010). In other situations, inhibition of autophagy promotes tumorigenesis (Qu et al., 2003). Therefore, autophagy may promote or inhibit tumorigenesis, depending on the cellular context.

Parkhitko and colleagues, using *TSC2*-null cells and animal models, found that the autophagy substrate p62/sequestosome 1 (SQSTM1) is a critical component of TSC driven tumorigenesis and that tuberin-null cells had decreased autophagy levels (Parkhitko *et al.*, 2011). The accumulation of P62/SQSTM1 in *Tsc2* ^*-/-*^ mouse embryonic fibroblasts (MEFs) initiates the development of TSC tumors. Indeed, accumulation of the autophagy substrate p62/SQSTM1 promotes tumorigenesis via activation of NF-κB and Nrf2 (Duran et al., 2008; Komatsu et al., 2010). Subsequently, autophagy cannot be induced adequately with numerous metabolic defects existing in *Tsc2* ^*-/-*^ MEFs and further suppression of autophagy may exhibit an inhibitory effect on tumor development, showing the paradoxical effect dependent on cellular metabolic status. Di Nardo and colleagues studied *Tsc2*-knockdown cells and found that neuronal TSC1/TSC2 complex allows autophagy by acting as a checkpoint on mTORC1 (Di Nardo *et al.*, 2014). Another study evaluated the mTORC1-dependent autophagy pathway in *Tsc2* ^*-/-*^ cells and observed a significant increase in MTORC1-mediated inhibitory phosphorylation of the autophagy initiating kinase ULK1 and reduced autophagosome formation. Treatment with rapamycin increased the LC3II/LC3I ratio but not total LC3 levels, showing that LC3 protein dosage would not be always useful to evaluate alterations in autophagy pathway (Pal et al., 2019). None of the studies above investigated heterozygous TSC cells to evaluate cellular processes before tumor formation. 

In Figure 3 (right), we can observe similar ratios between patient 1 and wild-type individual 6, while other patients had higher ratios and the wild-type individual 7 had lower ratios. This difference may be due to different mutations in *TSC1* or *TSC2* genes, which could lead to different levels of autophagic alterations. Patient 1 presents the less severe symptoms between the five patients included in this study. In literature, there are no studies evaluating the role of different types of mutations in *TSC1* and *TSC2* genes in the response to mTOR inhibitors. 

Finally, it is worth mentioning that in addition to tumor formation and patient variability in tumor spectrum, TSC patients may present a variety of non-tumoral symptoms, including seizures and complications in the central nervous system. Zhang et al. (2016) found that Tsc1 haploinsufficiency is associated with increased dendritic complexity and total dendritic length as well as increased Filamin A levels in the olfactory bulb of Tsc1 heterozygote mice. This could contribute to a spectrum of cognitive or psychiatric disorders in TSC patients (Zhang *et al.*, 2016). Recently, Haji and colleagues investigated the consequences of heterozygous knockout of Tsc1 in hippocampal medial ganglionic eminence cells and found impairment in their spatial working memory and a decrease in synaptic inhibition of pyramidal cells. This may also contribute to cognitive deficits in the Tsc1 mouse model of TSC (Haji *et al*., 2020). The findings in central nervous system using heterozygous models reinforce the need for such studies to evaluate TSC tumor formation. 

Although the present study has several limitations, we showed by a previously validated method that autophagy alterations are present in non-tumoral cells in patients with TSC. Additional studies at the protein level would be useful to confirm our findings. We focused on mTOR-dependent mechanisms, and further analysis of mTOR-independent mechanisms would be interesting. The autolysosome formation is the best way to evaluate autophagy alterations, which was performed in this study. However, additional studies, such as autophagic flux experiments and expression analysis of autophagy related proteins, are important and should be performed to confirm the findings described here. Moreover, Lombardi and colleagues have shown that rapamycin influences mitochondrial function in pancreatic cells (mitochondrial respiration and mitochondrial Ca^2+^ uptake in β cells) (Lombardi *et al.*, 2017). This could have a potential implication in mitophagy, which could be evaluated in future studies with TSC cells and rapamycin. Additionally, other mTOR regulated processes, like immune response and senescence could also be evaluated.

## Conclusions

Our results suggest that normal appearing cells which do not present tumor characteristics have autophagy alterations that are controlled by rapamycin treatment. This dysregulation in normal cells suggests that autophagy could be a mechanism that leads to tumor formation, before the second hit mutation and cell cycle alterations. Also, alterations founded in healthy portions of TSC skin provide an explanation to such a variety of lesions observed in this condition. Further studies using normal and tumor cells are needed to confirm our findings and would guide additional investigations about the mechanisms of tumorigenesis in TSC, and development of novel therapeutic options. Autophagy could be a target to therapy for TSC patients and possibly prevent or reduce tumor occurrence. Finally our findings also highlight the importance of conducting studies with heterozygous TSC cells to better understand the role of haploinsufficiency in the disease.

## Supplementary material

Table S1 - Number of acridine orange positive cells for each condition, patients and controls.
