# Supplementary material for: Primary cells derived from Tuberous Sclerosis Complex patients show autophagy alteration in the haploinsufficiency state
Source: Genet Mol Biol. 2021 Oct 1;44(4):e20200475. doi: 10.1590/1678-4685-GMB-2020-0475 (PMC8515215; doi:10.1590/1678-4685-GMB-2020-0475)
Supplement: Table S1 - [file 1415-4757-GMB-44-4-e20200475-s1.pdf]

Genetics and Molecular Biology
https://doi.org/10.1590/1678-4685-GMB-2020-0475

Supplementary Material to “Primary cells derived from Tuberous Sclerosis Complex patients show autophagy alteration in the haploinsufficiency state”

Table S1. Number of acridine orange positive cells for each condition, patients and controls.

	% acridine orange positive						
	**Patient 1**	**Patient 2**	**Patient 3**	**Patient 4**	**Patient 5**	**Control 1**	**Control 2**
DMSO N1	9,461249	2,9604	6,680295	9,854015	8,848353	5,023364	9,317682
RAPAMYCIN N1	6,96164	3,057792	21,199968	23,133039	17,126802	5,740014	0,830281
RAPAMYCIN/DMSO N1	0,736	1,0328	3,1735	2,3475	1,9355	1,1426	0,0891
DMSO N2	4,01493	0,744442	6,869681	6,68037	5,637379	8,954321	8,920426
RAPAMYCIN N2	3,482035	1,893708	9,110386	7,996406	7,302521	9,835082	1,806084
RAPAMYCIN /DMSO N2	0,867	2,5437	1,3261	1,197	1,1179	1,0983	0,2024
DMSO N3	8,676352	18,141324	8,203362	9,67581	6,914239	8,901629	7,831806
RAPAMYCIN N3	14,038964	23,8824	7,701687	10,184074	11,160714	9,768977	7,992417
RAPAMYCIN /DMSO N3	1,618	1,3164	0,9388	1,0525	1,6141	1,0974	1,0205
Mean ± DP (RAPAMYCIN /DMSO)	1,07 ± 0,48	1,63 ± 0,80	1,81 ± 1,19	1,53 ± 0,71	1,62 ± 0,32	1,11 ± 0,03	0,44 ± 0,51
